# Intensifying urban imprint on land surface warming: Insights from local to global scale

**DOI:** 10.1016/j.isci.2024.109110

**Published:** 2024-02-05

**Authors:** Pengke Shen, Shuqing Zhao

**Affiliations:** 1 National Climate Center, China Meteorological Administration, Beijing 100081, China; 2 College of Ecology and the Environment, Hainan University, Haikou 570228, China

**Keywords:** Earth sciences, Climatology, Environmental science, Global change, Remote sensing

## Abstract

Increasing urbanization exacerbates surface energy balance perturbations and the health risks of climate warming; however, it has not been determined whether urban-induced warming and attributions vary from local, regional, to global scale. Here, the local surface urban heat island (SUHI) is evidenced to manifest with an annual daily mean intensity of 0.99°C–1.10°C during 2003–2018 using satellite observations over 536 cities worldwide. Spatiotemporal patterns and mechanisms of SUHI tightly link with climate-vegetation conditions, with regional warming effect reaching up to 0.015°C–0.138°C (annual average) due to surface energy alterations. Globally, the SUHI footprint of 1,860 cities approximates to 1% of the terrestrial lands, about 1.8–2.9 times far beyond the urban impervious areas, suggesting the enlargements of the imprint of urban warming from local to global scales. With continuous development of urbanization, the implications for SUHI-added warming and scaling effects are considerably important on accelerating global warming.

## Highlights

• Surface urban heat island intensifies from 0.99°C to 1.10°C globally for 2003–2018
• Time can be substituted by space in predicting urban warming and energy alterations
• The imprint of urban warming through time enlarges from local to global scales
• Tailored strategies are required for adapting to the rapidly urban-induced warming

## Graphical abstract

**Figure:**
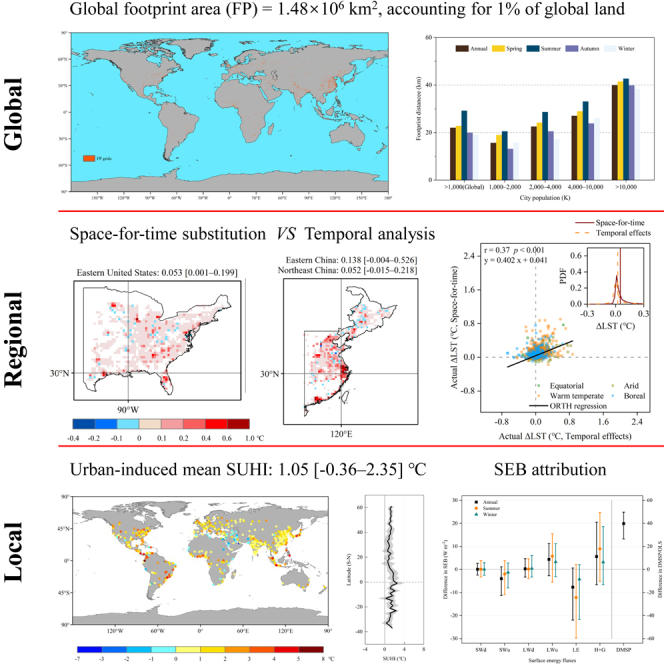

## Introduction

Urban environmental changes (such as atmospheric CO_2_ increase and urban heat island) are known as the harbingers of the future global change.^1^^,^^2^ Evidence suggests that global asymmetric warming (stronger trend in daily minimum temperature compared to the maximum) was stronger and observed decades earlier in urban areas.^3^ Typical urban climate effects—local urban heat island (UHI),^4^^,^^5^^,^^6^ regional-scale warming,^7^^,^^8^ and global greenhouse effect^9^—has been extensively reported, highlighting urban areas as hot spots in driving multiple climate changes.^8^^,^^10^ Although urban land cover is relatively little compared to Earth’s surface now,^11^ into the future, however, urban land cover will increase by 1.2 million km,^2^ tripling that circa 2000^11^ and urban population is projected to be over 5.17 billion (60.4% of the world’s population) by 2030.^12^ Human activity has changed the Earth’s climate markedly,^13^^,^^14^ in urban system in particular where confounding climate forcings imposed by both natural and anthropogenic factors associated with urbanization.^15^ Understanding urban-induced surface warming and attributions is crucial for quantitative assessment of potential thermal risk,^16^^,^^17^^,^^18^ formulating practical mitigation and adaptation strategies,^19^^,^^20^ as well as building holistic views of human impact on the Earth’s climate.

Research on UHI effects is overall subject to observational techniques. Canopy temperature measured by meteorological sensors promoted early UHI studies,^21^^,^^22^ whereas uncertainties exist on UHI intensity due to restricted geographic coverage, meteorological station migration, and definition of urban/rural.^7^^,^^23^^,^^24^ Refinement of the urban climate models from one-to three-dimensional mesoscale, has complemented air UHI and contributed to boundary layer UHI research.^19^^,^^25^^,^^26^ Climate simulations are often constrained by coarse spatial resolution, predefined scenarios and uncertain physical parameterization,^26^^,^^27^ and tremendous computation for global extent.^28^ Rapid advancements in satellite remote sensing and abundant data since 1972 (the first satellite-based UHI report) have considerably boosted the surface UHI (SUHI) studies,^29^^,^^30^ particularly in the last decade,^6^^,^^8^^,^^31^^,^^32^^,^^33^^,^^34^ owing to high spatial resolution and wide coverage, temporal synchronization/short-period observation on land surface temperature (LST) and so forth.

Despite the diversity of observation and modeling systems, urban imprint on warming magnitude or contribution has been controversial across varying spatial scopes and counties/regions.^7^^,^^35^^,^^36^^,^^37^^,^^38^ Previous studies revealed that urban warming influenced around one-third of observed warming on average in mainland China^7^^,^^24^ yet with heterogeneities associated with vegetation activity or other land use variations.^28^^,^^39^ Other studies highlighted a small occupation of urban areas (for example, less than 1% of China’s land mass) may result in negligible influence on large-scale warming.^3^^,^^40^ IPCC^14^^,^^41^ extrapolated that urban warming would likely contribute up to 10% to centennial averaged temperature trends globally; however, in some regions with rapid urbanization (e.g., urban agglomerations), impacts on large-scale warming could be substantially larger,^23^^,^^42^ given the footprint (FP) of UHI effect largely exceeding urban boundary.^43^^,^^44^ Zhou et al.^8^ and Shen et al.^45^ provide global-scale satellite assessments of hypothesized potential or actual warming due to urban expansion based on spatial gradient model. However, the understanding of how urbanization process impacts three cross-level scales (i.e., local SUHI effect, regional warming magnitude, global FP area) is still disputed;^14^^,^^17^^,^^40^ Besides, quantitative driving attribution analyses combined energy balance perturbations on the multi-spatial scales are critical for formulating strategies in urban adaptation.

Here, we use high-resolution annual maps of global artificial impervious area (GAIA), MODIS land temperature, and related radiative and energy fluxes (including nighttime light, NTL) to systematically disentangle urbanization effects on multiple spatial scales from local (SUHI intensity on 536 large cities), regional (warming magnitude in nine major urbanized regions) and global (spatial footprint area) scopes specifically. We focus in particular on the spatial-temporal pattern of SUHI, driving attributions (in terms of surface energy balance, SEB), potential and actual warming magnitudes, spatial and temporal sensitivities of warming, and the footprint estimation of SUHI effect, for addressing warming effects induced by urbanization locally to globally.

## Results

### Spatial and temporal patterns of local SUHI effect and attribution

**Figure 1:**
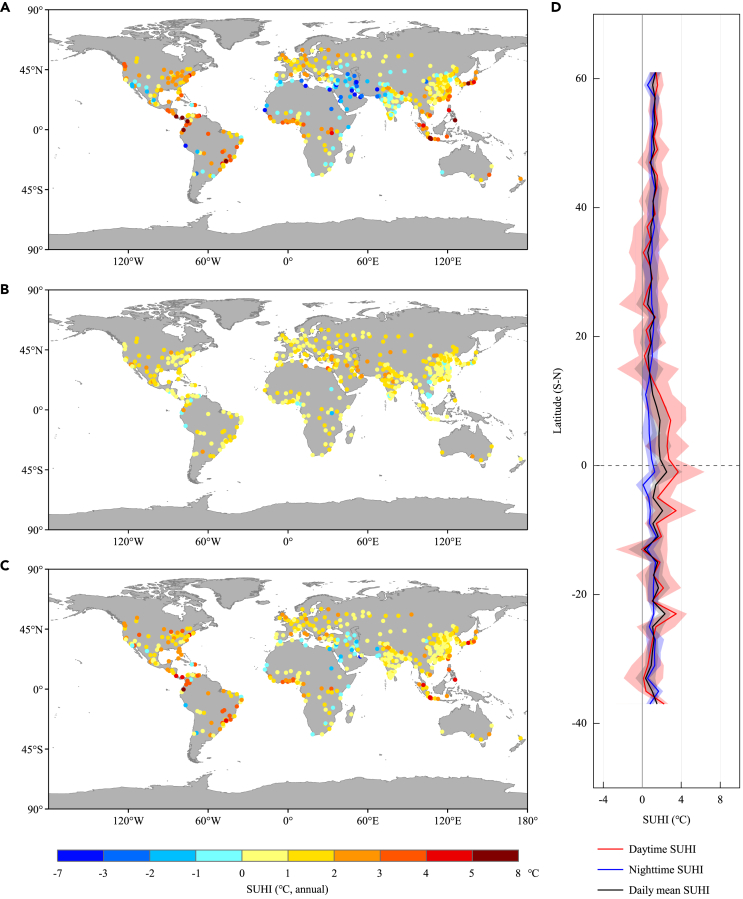
Distributions of annual SUHI intensity averaged over the period 2003−2018 across global 536 large cities (A) Daytime SUHI. (B) Nighttime SUHI. (C) Daily mean SUHI (unit: °C). (D) shows the latitudinal average of SUHIs at annual scale, solid lines and shaded areas denote the mean and 5%–95% range, respectively.

SUHI intensity varies widely across global large cities (Figures 1, S1, and S2). Daytime SUHI has great spatial heterogeneity with a mean [5–95% range] of 1.14 [−1.86–3.79] °C. The extremely strong SUHI (more than 6.0°C) occurs in San José (Costa Rica) and Quito (Ecuador); while negative SUHI is generally located on the Arabian Peninsula and its East, and parts of India. SUHI in nighttime presents positive intensity consistently with 0.97°C on average. By comparison, most of the cities, and especially the larger ones, exhibit positive in daily mean SUHI and diurnal temperature range (DTR) of SUHI. We also note that for the temporal variation (Figure S3), estimates of SUHI increases over year, e.g., 1.05°C–1.25°C at daytime and 0.99°C–1.10°C for daily mean, and possess seasonal asymmetry (the strongest in summer and the weakest in winter). There are significant correlations between summer and winter SUHI, but no correlation between daytime and nighttime, indicating disparate driving mechanisms of SUHI between day and night.

**Figure 2:**
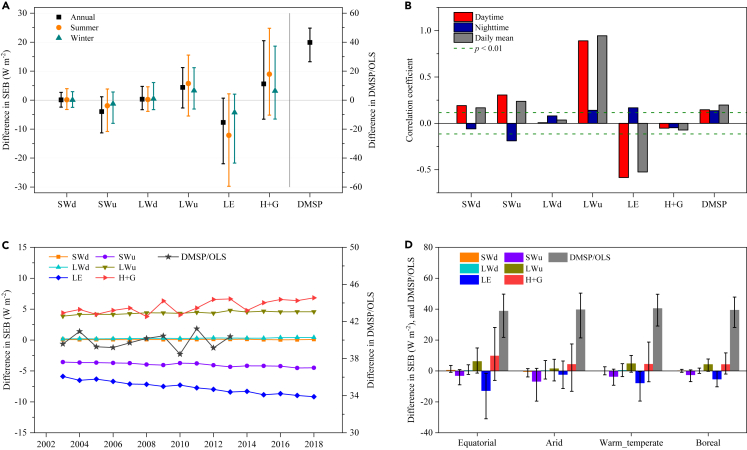
Attribution analysis on spatial SUHI intensity, in terms of surface energy alteration for 536 large cities (A) Difference in SEB flux between urban and rural areas, averaged by all large cities. (B) Spatial correlation coefficients between SUHI (in daytime and nighttime) and the difference in SEB fluxes between urban and rural areas. (C) Temporal variations of the difference in fluxes. (D) Difference in SEB and DMSP/OLS among major climate zones (i.e., equatorial, arid, warm temperate, and boreal zones).

Attribution of SUHI are quantified for 536 large cities on the basis of corresponding SEB variations (Equation 2) in urban areas compared to the surrounding countryside (Figures 2 and S4). The overall differences in downward radiation (SWd and LWd) between urban and rural are near zero. Most of cities show decline trend in SWu (88.4%) and LE (91.6%) in urban areas, with a mean [5–95% range] of −3.97 [−11.31–1.16] W m^−2^ and −7.70 [−21.97–0.67] W m^−2^ at annual scale, respectively. Contrarily, differences in the LWu and (H + G) are basically positive. Increased DMSP/OLS in all large cities (39.80 [26.47–49.72]) demonstrates great contribution of anthropogenic heat to SUHI. Spatially, both daytime and nighttime SUHIs are correlated significantly with several SEB terms, in particular, daytime SUHI and differences in SWu, LWu (r = 0.31, 0.89; p < 0.001), and LE (r = −0.59, p < 0.001), nighttime SUHI and difference in SWu (r = −0.19, p < 0.001) (Figure 2B). Similar correlations of SUHI and SEB fluxes in space can be observed at seasonal scale, although with variable magnitude (Table S1), suggesting that geographic discrepancies across locations rather than seasonal variation of SEB, shape spatial pattern of SUHI in large cities globally. Temporally, our findings reveal that on the whole, SWu and LE variations decrease from −3.56 and −5.88 W m^−2^ to in 2003 to −4.49 and −9.17 W m^−2^ in 2018 respectively (Figure 2C), which dominate the strengthening of SUHI over year (Figure S3A). Consistent conclusions are detected when using fixed urban and rural pixels of 2003.

The SUHI intensity is influenced strongly by climate-vegetation background. For example, daytime SUHI is the most apparent in equatorial climate, followed by warm temperate and boreal climates, while in arid climate it exhibits cold island effect (Figure S5). During the night, SUHI presents stronger in arid and boreal climates than others. The changes of LE and SWu reveal negative in urban areas, yet with variable magnitude across all climates, dominating SUHI in daytime and nighttime, respectively. However, positive alteration in H + G plays compensatory effect that suppresses SUHI intensity across climates (Figure 2D). On the other hand, SUHI intensity also varies with vegetation coverage, especially in daytime (Figure S6). It is noteworthy that locations with lower NDVI (<0.4) where urbanization leads to cool island effect and diminishes DTR. Variations in SEB reports that decreased albedo (SWu) plays dominant role of cool island in vegetation deficient zones (NDVI <0.4). Whereas, decline in LE becomes the main driver of SUHI effect in medium and high vegetation cover zones.

### Potential and realistic warmings in major urbanized regions

**Figure 3:**
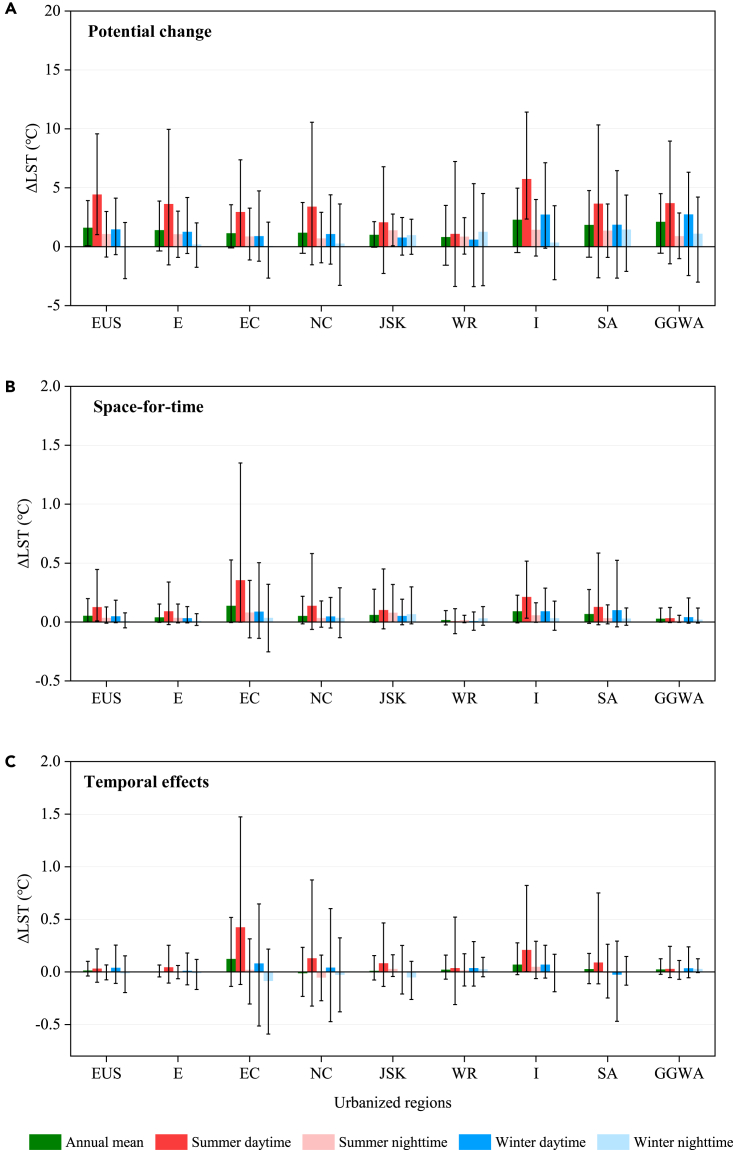
Changes in annual and seasonal LSTs caused by urbanization in major urbanized regions, spanning 2003–2018 (A) Potential change. (B) space-for-time. (C) temporal effects. Histogram and error lines represent the mean and 5–95% range. Abbreviations on X axis represent the nine major urbanized regions across the globe, i.e., Eastern United States (EUS), Europe (E) Eastern China (EC), Northeast China (NC), Japan and South Korea region (JSK), Western Russia (WR), India (I), Southeast Asia region (SA), and Gulf of Guinea, West Africa (GGWA), respectively.

Potential change in regional LST (Figures 3A and S7) is obtained by space-for-time estimation assuming increased ISP from 0 to 50%. Annual ΔLST ranges from 0.82 [−1.57–3.50]°C to 2.29 [−0.50–4.96]°C across the nine major regions. ΔLST exhibit positive across most locations in Eastern United States, Europe, Eastern and northeast China. In summer daytime potential ΔLST is generally the strongest across regions compared with other seasons, e.g., in winter daytime and nighttime. Then actual warming magnitude (Figure 3B) is estimated by the product of space-for-time outcome (when ISP increases 1%) and actual ISP increment during 2003–2018 (Figure S8). Non-linear growth (e.g., quadratic and cubic curves) of ISP across most locations has been detected. ISP growth in Eastern China (6.6 [0.8–17.3] %) is significantly higher than in other regions (0.7–2.6 [0.1–9.0] %). As such, actual warming magnitude in the former region is strongest (e.g., 0.138 [−0.004–0.526]°C at annual scale) (Figure S9). It is followed by Japan and South Korea region, and Southeast Asia region (0.068–0.092 [−0.011–0.276] °C). The India region has the lowest warming on average (0.015 [−0.025–0.097] °C) due to negative estimations on some locations. The actual warming magnitude is consistently stronger in summer (and in daytime) than in winter (and in nighttime) for these regions.

Further, temporal effects of urbanization have been quantified by isolating urban-induced warming signal from natural variability. As expected, most of the urbanized regions, and especially in Eastern China, witnesses pronounced urban-induced warming (0.004–0.123 [−0.139–0.519] °C) over the 2003–2018 period, except in Northeast China where cooling effect happens (Figures 3C and S10). In Eastern United States and Europe, however, temporal warming caused by urbanization shows relatively lower than the space-for-time estimation due to several locations with negative trends detected. Furthermore, the character of temporal asymmetries for seasons and daytime/nighttime in most regions embodies in accordance with space-for-time estimation.

**Figure 4:**
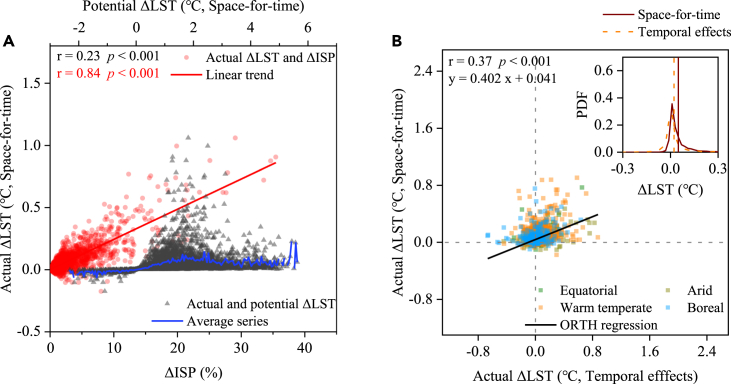
Comparisons in potential and actual warming in the nine urbanized regions worldwide during 2003–2018, according to space-for-time substitution and temporal analysis (A) Relationships between actual ΔLST and ΔISP, potential ΔLST, with linear analysis, average series, and Pearson’s correlation coefficient. (B) Scatterplot of in actual ΔLSTs, including four major climates (equatorial, warm temperate, arid, and boreal zones), between the two approaches. The linear fitting in (B) is based on an orthogonal or total least squared regression procedure (ORTH) regression, with Pearson correlation test. The small graph in the upper right corner shows the probability distribution function (PDF) of annual ΔLSTs by two estimations.

To further contextualize the estimates, Figure 4 displays the relationships between actual ΔLST and ΔISP, potential ΔLST and consistency through two approaches. The actual warming is determined by both potential ΔLST and ΔISP during study period, whereas increment in ISP seems to contribute largely to actual ΔLST owing to higher correlation between actual ΔLST and ΔISP (r = 0.84, p < 0.001) compared to actual and potential ΔLST. The interpolations of the orthogonal or total least squared regression procedure (ORTH) regression demonstrate that a significant linear dependence relation (with rate of 0.402, r = 0.37 and p < 0.001) for realistic ΔLST quantified by two methods. And the probability density functions (PDFs) of ΔLST show that space-for-time substitution generates more concentrated distribution of estimations, but relatively higher average values than temporal impacts of urbanization. Nevertheless, the urban-induced LST warmings of the same order of magnitude at regional scale are overall corroborated by space-for-time and temporal analysis.

Potential and actual warmings are attributed to perturbations of SEB fluxes at regional scale (Figures S11–S13). First, a reduction of LE, especially during summertime, dominates potential warming in all major regions. SWu presents decline in part of regions (e.g., Eastern China, Western Russia, Southeast Asia region) and changes across seasons which are determined by albedo comparisons. ΔLWu and Δ(H + G) are positive at annual scale in general for all regions. In winter, however, negative Δ(H + G) plays a more important role in regional warmings. Second, regarding actual warmings of urbanization, the reduction in LE with estimations of −1.56–0.20 W m^−2^ (space-for-time estimate) and −1.44–0.05 W m^−2^ (temporal analysis) contributes primarily to all urbanized regions. Likewise, a leading role of LE exhibit more evident in summer season. Other flux such as ΔSWu and Δ(H + G) estimated by two methods witness positive or negative perturbation which varies across regions and seasons. In addition, ΔDMSP/OLS that reflects anthropogenic heat emissions embodies positive in these regions according to temporal analysis.

### Global impacts of urbanization on warming trend and spatial extent

**Figure 5:**
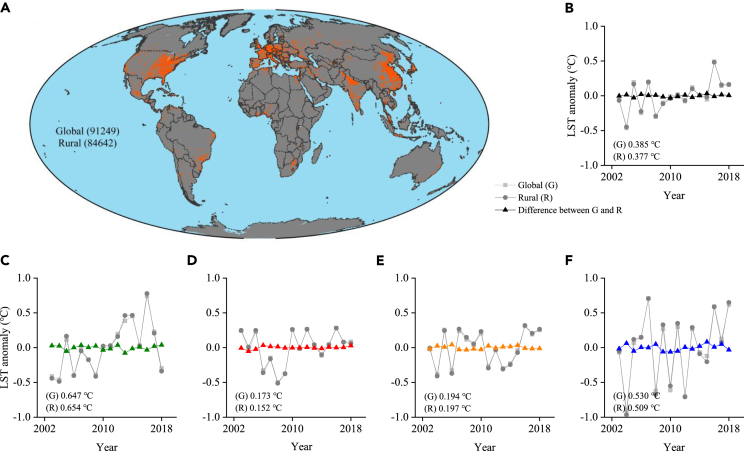
Global and rural mean LST anomalies as well as their difference over the period 2003–2018 (A) Global rural grid points on 0.5° raster grid. Gray and red colors denote global land grid points, where gray denotes rural grid points. (B–F) Annual mean, spring, summer, autumn, and winter, respectively. The numbers on the plots denote the temperature trend over 16 years (unit: °C).

The area of global urban impervious surfaces has increased from 5.07 × 10^5^ km^2^ (0.34% of total land surface) in 2003 to 8.02 × 10^5^ km^2^ (0.54%) in 2018; The impervious area on each continent in descending order is: Asia (1.76 × 10^5^–3.29 × 10^5^ km^2^, 2003–2018), North America (1.60 × 10^5^–2.17 × 10^5^ km^2^), Europe (0.95 × 10^5^–1.45 × 10^5^ km^2^), Africa, South America, and Oceania (Figure S14). On the basis of ISP threshold (<1% on 0.5° raster grid), we separate rural raster (with number of 84,642) from global land surface (91,249) (Figure 5A). By comparison, the climatic trends of global and rural land LST are consistent in the period 2003–2018 (0.385 vs. 0.377°C) with slight deviation (S.D. = 0.018°C) at annual scale. Comparisons across seasons are as follows: spring (0.647 vs. 0.654°C, S.D. = 0.016°C), summer (0.173 vs. 0.152°C, 0.009°C), autumn (0.194 vs. 0.197°C, 0.011°C), and winter (0.530 vs. 0.509°C, 0.021°C) (Figures 5B–5F). It suggests that a weaker ‘bias’ between global and rural LST series (one order of magnitude smaller) is due to urbanization or possibly influenced by climate variability across locations. Overall, global urban-induced LST bias could be a small fraction of climate change signal observed by remote sensing. At annual scale, the well-known global surface warming is not significantly impacted by urban warming.

**Figure 6:**
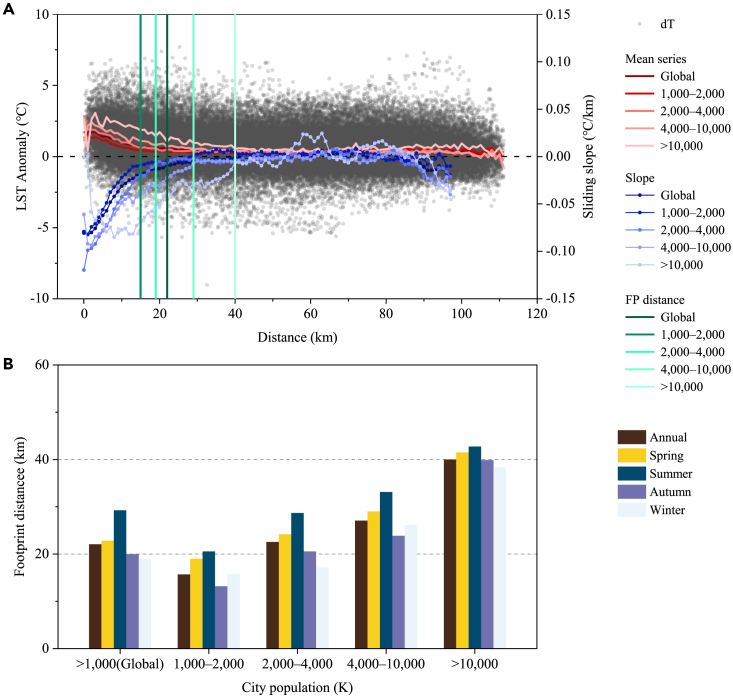
The footprint (FP) of SUHI for global 536 large cities at annual and seasonal scales (A) Annual FP analysis in 2018. The black dots denote the LST anomaly (left Y axis, °C) and the distance of the grid point (0.05°) from the urban center distance (X axis, km); red line represents the mean series of LST anomaly calculated by each 1 km interval, the blue line is the sliding trend of the mean series (right Y axis, °C/km); and the vertical solid line represents the FP distance. (B) Annual and seasonal FP values averaged during period 2003–2018. Global 536 large cities are classified as four categories based on city population 1,000–2,000 K (number is 282), 2,000–4,000 K (147), 4,000–10,000 K (74), and 10,000–40,000 K (33).

The FP of SUHI effect has been discovered globally (Figures 6 and S15). In 2018, for instance, mean LST anomaly tends to zero with the distance extending outward from urban center for 536 large cities worldwide. The trend of sliding LST series declines down to 0.01°C/km (absolute value) where distance equals to 22 km (defined as FP value). We also find that FP value enlarges dramatically with higher population gradients of large cities: PF = 15 km (1,000–2,000 K), 19 km (2,000–4,000 K), 29 km (4,000–10,000 K), and 40 km (>10,000 K). Moreover, FP distance varies across seasons, e.g., the highest occurs in summer (on average, 29.3 km for 2003–2018), followed by spring (22.8 km) and autumn (20.0 km), while FP in winter is relatively low (19.0 km), corresponding to the seasonal SUHI variations described in the first section.

Based on the FP analysis above, we conduct a global maximizing estimation of FP area for 1,860 cities (greater than 300 K population in 2018) (Figure S16; Table S2). At annual scale, FP area reaches 1.48 × 10^6^ km^2^ that covers around 1.00% of the global land surface; FP area and the percentage across seasons are 2.45 × 10^6^ km^2^ and 1.64% (spring), 2.99 × 10^6^ km^2^ and 2.01% (summer), 1.17 × 10^6^ km^2^ and 0.78% (autumn), 1.44 × 10^6^ km^2^ and 0.97% (winter), respectively. Asia has the largest FP area (annual scale, 8.22 × 10^5^ km^2^, 1.87%) apparently owing to a high proportion of cities, followed by North America and Europe (1.90 × 10^5^ km^2^, 0.79%; 1.75 × 10^5^ km^2^, 1.75%). Despite little impact of FP area of SUHI effect in space domain at the globe land, the area exceeds significantly the coverage of urban impervious surfaces, around 1.8–2.9 times the later at annual scale, and in summer the ratio is up to 3.7–5.8 times in 2003–2018.

## Discussion

The City Clustering Algorithm^46^^,^^47^ as well as threshold definition for rural pixels provides scientific estimates in SUHI intensity which avoids the interference deriving from FP influence.^43^^,^^44^ In daytime, local SUHI effects of global 536 cities show a great spatial heterogeneity (Figure 1), coinciding with previous studies (e.g., distribution, cold island).^27^^,^^34^^,^^48^ Using dynamic boundary delineation for urban/rural areas, we demonstrate that most cities estimate reductions in SWu (albedo factor)^49^^,^^50^ and especially in LE,^51^ and increased anthropogenic heat emissions,^52^ largely contributing to SUHI formation in daytime and nighttime (owing to storage heat during the daytime for a later release).^33^^,^^48^^,^^53^ Temporally, SUHI enhances persistently (1.05°C–1.25°C, daytime) for the 2003–2018 period due primarily to decreasing trends in both SWu and LE (Figure 2). Non-contradictory spatiotemporal attributions are found across locations, suggesting the effectiveness of albedo management, urban planning and landscape irrigation for thermal adaptation to urban environments.^19^^,^^52^

There is growing evidence that the magnitude of SUHI can be strongly explained by climat^31^^,^^38^ and urban-rural greening condition.^54^ According to our estimates, SUHI effect in the equatorial belt, particularly for summer daytime, can be attributed to a typically greater loss of latent heat cooling through evapotranspiration, which is line with existing attribution studies on this topic.^32^^,^^51^ One study reports that future climate risk is projected to be greater in cities located closer to the equator^55^ as economic resources for mitigating climate change are generally more limited.^56^ However, in arid regions, the intensity of daytime SUHI is often negative as opposed to nighttime, which is due primarily to decreased urban albedo, enhanced sensible heat dissipation and heat storage (greatly contributes to nighttime SUHI as reduced albedo in urban areas, Figures S4 and S5; ref.^48^^,^^49^) as well as anthropogenic thermal emission. Additionally, human interventions in the city—irrigation of lawns, evaporation from open aqueducts, etc.— contribute to daytime LE enhancement and cooler effects,^32^ which may counteract the warming caused by increased impervious surfaces. Other evidence shows sharp variations of SUHI intensity across NDVI gradients (Figure S6), indicating the implementation of more effective tree planting and irrigation in urban environments could offer an immediate way to mitigate urban-induced warming related to urban population exposure.^57^ However, alleviating warming and available water resources are closely coupled (synchronous occurrence) in arid cities,^58^ requiring strong trade-offs to be resolved,^31^ as water scarcity would be exacerbated as a result of future climate changes.^59^

Urban-rural gradient models on urbanization effects are utilized extensively in recent years.^8^^,^^32^^,^^45^ The methodology offers prominent advantages when presenting spatial variation modes of climatic or environmental elements, as well as predicting the consequences of future urbanization. We find that regional LST increases of up to 0.092°C, 0.034°C (annual daytime and nighttime) averaged by global nine urbanized regions from 2003 to 2018 (Figure 3A). We disclose the fact of non-linear growth (e.g., quadratic and cubic curves) of urban impervious areas and ISP during the past decades,^60^ which makes it difficult to estimate the realistic urbanization effects, because regional-scale warming generally contains both natural climate variability and urbanization signal.^24^^,^^35^ In this regard, we compare results from both space-based approach and a temporal scheme we designed via isolating urban-induced warming signal^42^ in parallel period 2003–2018. Consistent magnitude deriving from the two estimations with significant ORTH correlation (r = 0.37, p < 0.01; Figure 3B) demonstrates the feasibility of space-for-time substitution^2^^,^^45^^,^^61^ in understanding and predicting urban imprint on surface temperature, which opens venues for potential future research given spatial data are generally easier to obtain than temporal data.

We decipher attributions on regional warming in urbanized regions by applying both space-for-time and temporal methods, suggesting that ISP increment (Figures 4A and S8) as well as concomitant perturbation of SEB synergistically promote realistic warming over the study period. We emphasize that reduction in LE at annual scale (a dominant factor, ref.^45^^,^^51^) and diametrically opposite H + G (i.e., ‘cooling’ effect), emitting LWu (in accordance to the fourth power of LST variation following Stefan-Boltzmann’s law^62^), variations in SW (depending on the disparities in albedo between urban and rural surfaces, ref.^49^^,^^50^), and positive DMSP/OLS (reflecting anthropogenic heat emissions), jointly regulate surface warming but vary across seasons and regions. For instance, the summer LE decline overwhelmingly drives surface warming especially in Eastern and Northeastern China, Japan and Korea regions, and Southeast Asia; In winter, however, alterations in LWu and H + G instead of LE play more critical roles in partial areas (Figures S12 and S13). These perturbations induced by urbanization are also associated tightly with other land use activities and regional climate background.^45^ It is thus imperative to improve tailored mitigation and adaptation strategies based on specific SEB attribution on the regional level given warmer climate in the future,^14^ considering also the strong differences in climatic conditions.^31^^,^^52^

To date the urban impervious coverage occupies less than 1% of world’s land mass (Figure S14; ref.^60^^,^^63^). However, it remains elusive regarding urban-induced warming impact on global scope due to the deficiency of optimal calculation and spatial coverage of ground observations such as meteorological stations^64^ and coarse spatial resolution in the atmospheric reanalysis datasets. By comparing rural and global LST series observed by satellite remote sensing, we derive conclusions that globally, LST changing trends (annually and seasonally) are not significantly impact (or ‘disturbed’) by urban warming during the recent decades. What’s more, assessments based on SUHI effect indicate that urbanization contributes little to global warming;^8^ for reference, our overall estimate that FP area of annual SUHI reaches 1.48 × 10^6^ km^2^, accounting for 1% of global land. Nevertheless, the area is far more than global urban impervious areas (around 1.8–2.9 times at annual scale), especially during summertime 2003–2018 (3.7–5.8 times) when the SUHI intensity is presented to be the strongest.^5^^,^^31^^,^^43^ We expect the ratios of global FP area growth to continue, and perhaps accelerate, in an urbanizing world, as SUHI enhance and projected impervious surface expansion proceed.^11^^,^^50^^,^^65^

We conclude that urbanization-induced surface warming effects, assessed at the multiple levels (i.e., local, regional, global), are typically subject to varying spatial scales. This confirms (with quantitative estimates as well as attribution discussion based on SEB equation) results from the latest studies that reported urbanization contributes little to global scale yet substantially intensifies local-to regional-scale warming.^8^^,^^42^ We also identify the human influence (via anthropogenic heat emissions) on SUHI-added warming in urban regions^13^^,^^15^^,^^66^ and the Earth’s climate system.^14^ The estimates of the urban imprint on surface warming fill an important knowledge gap in cities and climate change of the Intergovernmental Panel on Climate Change (IPCC) special report, and hence help to further increase confidence in understanding and predicting ongoing urbanization effects. Furthermore, the observed urban warming and SEB attribution across spatial scales highlight the importance of using judicious and tailored urbanization strategies that consider different climatic contexts to adapt to future global climate change.

### Limitations of the study

Some limitations of this study should be acknowledged. Despite the quantitative evaluations of urban-induced warming of this study, it has been difficult to empirically distinguish complex connections or boundaries among the multiple spatial scopes. However, it is safe to state that the changing impacts (magnitude, scope) of urban warming with expanded spatial scales—from local (for example, annual SUHI: 1.05°C, FP = 22 km, Figures 1C and 6B), regional (0.015°C–0.138°C, Figures 3B and S9), to global (FP covers only 1%, Figure S16; Table S2)—has been discovered. Additionally, discrepancies in spatial-temporal estimates of urbanization effects exist, due probably to differential temperature sensitivities in space and time^2^ in diverse climatic contexts^31^^,^^45^ as well as possible influence of rural agricultural activities on urban heating effects.^67^ We also make clear that space-for-time logic for predicting temporal change may be inherently limited when cities are sprawling rapidly and non-linearly, and that diverse types and characteristics of urban landscapes need to be further considered.^13^ Another limitation is the combination of sensible heat and ground heat fluxes (i.e., H + G),^39^^,^^68^ which makes it unable to furtherly dissociate surface-atmosphere interaction mechanisms.^69^ Equivalent high-resolution observations of canopy air temperature and wind velocity combined with a bulk aerodynamic equation (to calculate H)^70^ would help address this dilemma.

## STAR★Methods

### Key resources table

**Table:** 

REAGENT or RESOURCE	SOURCE	IDENTIFIER
**Deposited data**		

MODIS LST and Emissivity data (MYD11C3), surface albedo (MCD43C3), and digital elevation product GMTED2010	United States Geological Survey (USGS)	https://earthexplorer.usgs.gov
MODIS latent heat flux (MOD16A2) and water body (MCD12C1) data	The Level-1 and Atmosphere Archive & Distribution System Distributed Active Archive Center (LAADS DAAC)	https://ladsweb.modaps.eosdis.nasa.gov/search
CERES radiation flux	National Aeronautics and Space Administration (NASA)	https://ceres.larc.nasa.gov/products-info.php?product=EBAF
DMSP/OLS nighttime light	National Oceanic and Atmospheric Administration (NOAA) National Centers for Environmental Information (NCEI)	https://www.ngdc.noaa.gov/eog/dmsp/downloadV4composites.html
GIMMS NDVI 3g.v1 data	National Oceanic and Atmospheric Administration (NOAA)	https://ecocast.arc.nasa.gov/data/pub/gimms/
Annual maps of GAIA	Peng Cheng Laboratory	https://data-starcloud.pcl.ac.cn/zh
World Urbanization Prospects 2018	United Nations	https://population.un.org/wup/
Re-analyzed Köppen-Geiger climate map	Climate Change & Infectious Diseases Group, University of Veterinary Medicine Vienna	http://koeppen-geiger.vu-wien.ac.at

**Software and algorithms**		

MATLAB 2019b	Mathworks	https://www.mathworks.com/products/matlab.html
ArcGIS 10.6	ESRI	https://www.arcgis.com/index.html
Origin 2021b	OriginLab	https://www.originlab.com/OriginProLearning.aspx

### Resource availability

#### Lead contact

Further information and requests for resources should be directed to and will be fulfilled by the Lead Contact, Shuqing Zhao (shuqing.zhao@hainanu.edu.cn).

#### Materials availability

This study did not generate new unique reagents.

#### Data and code availability

• This paper analyzes existing, publicly available data. These accession numbers for the datasets are listed in the key resources table.
• This paper does not report original code.
• Any additional information required to reanalyze the data reported in this paper is available from the lead contact upon request.

### Method details

#### Data sources

##### Satellite remote sensing data

We collect monthly LST data across the globe during January 2003 to December 2018 from the Moderate-Resolution Imaging Spectroradiometer (MODIS) satellites Aqua (MYD11C3v006). LST data are measured under clear sky conditions at local time ∼1:30 AM and 13:30 PM (when passing the equator from south to north). MYD11C3v006 also provides monthly Emissivity including the bands of ε_29_ (8,400–8,700 nm), ε_31_ (10,780–11,280 nm), and ε_32_ (11,770–12,270 nm) on 0.05^o^ Climate Modeling Grid (CMG). Surface albedo from MCD43C3 (black-sky and white-sky) and latent heat (denoted as LE) from MOD16A2 with 8-daily estimates during 2003-2018 are used for the calculation of SEB equation. Yearly water body (2003-2018, 0.05^o^) deriving from MCD12C1 based on International Geosphere-Biosphere Programme (IGBP) are applied for SUHI calculation by excluding grids with more than 60% of water.

Surface radiative flux (downward and upward long- and short-wave fluxes for all-day and clear-sky conditions) are provided by NASA CERES (Clouds and the Earth's Radiant Energy System) Energy Balanced and Filled (EBAF)-Surface v4.1^71^ with resolution of 1^o^ globally, spanning 2003-2018. Version 4 Defense Meteorological Satellite Program/Operational Linescan System (DMSP-OLS) provided by NOAA Earth Observation Group shows light intensity ranging from 0–63 with resolution of 30 arc second grids globally, spanning 2003-2013.

For the sake of SUHI calculation, the GMTED2010 digital product (Global Multi-resolution Terrain Elevation Data 2010; ref.^72^) with 30-arc-second resolution is obtained to remove elevation influence in urban/rural areas. We also collect the GIMMS (Global Inventory Modeling and Mapping Studies) NDVI (normalized difference vegetation index) 3g.v1 data set with 0.083°×0.083° and 15-day composite from 2003 to 2015 to disentangle vegetation impact on SUHI distribution patterns.

##### Urban population and impervious surface map

The Population Division of the Department of Economic and Social Affairs of the United Nations has been issuing for several decades revised estimates and projections of the urban populations of all countries in the world. The newly released 2018 Revision of World Urbanization Prospects summarized 1,860 cities where over 300,000 urban population live.^12^

The high-resolution Global Artificial Impervious Areas (GAIA) has evident advantages in SUHI effect and urban climate research^73^^,^^74^ since the replacement of natural surfaces by impervious materials (e.g., streets, buildings, roofs) due to human activities is one of the most typical features of urbanization, which results in comparisons of energy and temperature in urban environments to their rural counterparts.^29^ We adopt annual maps of GAIA with 30-m resolution from 1985 to 2018 based on the Finer Resolution Observation and Monitoring of Global Land Cover (FROM-GLC),^60^ then aggregate them to impervious surface percentage (ISP) on global 0.05^o^ raster.

##### Köppen-Geiger climate

Since previous studies have emphasized the significance of background climate in modulating the SUHI intensity,^31^^,^^52^ we also investigate the relationship between variations in SUHI and re-analyzed Köppen-Geiger climate zone.^75^ The map contains five major climates encompassing equatorial, arid, warm temperate, boreal, and polar zones.

#### Calculation of SUHI intensity and surface energy balance

Existing definitions of SUHI intensity use administrative or physical boundaries (e.g., non-dynamic urban boundary based on impervious surfaces or vegetation index through satellite remote sensing) and certain buffer zones as rural references, leading to significant biases in estimation owing to the footprint of SUHI effect^43^^,^^44^ and accelerated urban expansion.^25^ To deal with this uncertainty generated by space-time dislocation, we extract dynamic boundaries in 536 large cities worldwide annually during 2003-2018 by taking into account demographic data (urban population is greater than 1 million in 2018) (Figure S1), the City Clustering Algorithm (CCA),^46^^,^^47^^,^^48^ and rigorous threshold definition for rural pixels (such as ISP < 5% on 0.05^o^, elevation difference is within 50 m, non-water bodies, the proximity principle, etc.) The boundaries obtained demonstrate great representatives of urban and corresponding rural references through comparisons with multi-period maps (i.e., 2005, 2010, 2015, 2018; ref.^76^). The detailed SUHI algorithm (Equation 1) for delineating urban and rural areas and definition of seasons are presented as follows.

(Equation 1) $$\text{SUHI}=\text{LS}T_{u}-\text{LS}T_{r}$$

Where LST_u_ and LST_r_ are the mean LSTs for all pixels within urban and rural areas, respectively.

Step 1. Regarding the urban areas, we first check the center location for each city (provided by UN data), and set the grid (0.05^o^) as initial pixel (denoted as *A*) of urban sequence (*U*) if the ISP is greater than 20%. There are 536 large cities in total worldwide detected and then identified as study objectives. Examine the eight neighbors around *A* for each city and add adjacent urban pixels with ISP exceeds 20% into the *U* sequence.^48^ Then retrieve the neighbors of the new effective urban pixels, and so forth. Search stops until no new eligible urban pixels (adjacent pixels, ISP ≥ 20%) are added.

Step 2. Regarding the rural references of cities, we obtain them on the basis of combination factors encompassing the ISP threshold (less than 5% on 0.05^o^ grids), altitude difference (within ± 50 m of the urban altitude range), and non-water bodies (based on International Geosphere Biosphere Programme, IGBP). A radius of 1^o^ around urban center is assumed to be the farthest boundary which avoids remote sampling of rural pixels for a given city. We detect the rural sequence (denoted as *R*) based on the proximity principle (outward from the city center) up to the spatial boundary or size of *R* sequence reaching twice corresponding *U* sequence.

We examine SUHI in daytime, nighttime, and daily mean, diurnal temperature range (DTR) of SUHI at annual and seasonal scales. Seasons were defined as follows: spring from March to May, summer from June to August, autumn from September to November, and winter from December to February in the northern hemisphere, and the southern hemisphere is just the opposite.

SUHI effect is essentially caused by reallocation of surface energy budget (represented by short/long-wave radiations, and sensible, latent, ground heat fluxes, anthropogenic heat) after the replacement of natural lands with urban structures like impervious surfaces.^29^ The typical surface energy balance (SEB) of urban surface is expressed as:

(Equation 2) $$\text{SWd}-\text{SWu}+\text{LWd}-\text{LWu}+Q_{A}=\text{LE}+H+G$$

In Equation 2, SWd, SWu, LWd, LWu are respectively the downward and upward radiative fluxes in the shortwave or longwave parts of the spectrum (that is, incident solar radiation, reflected solar radiation, incoming longwave radiation, outgoing longwave radiation). LE, the latent heat flux, is the exchange of energy between the surface and atmosphere that occurs when water is evaporated from or condenses onto the surface;^77^ and the combination of H + G (due to the scarcity of high-resolution data as well as calculation, ref.^39^^,^^53^) represents composed of sensible heat (which is the exchange of energy between the surface and atmosphere that results from the temperature difference) and ground heat fluxes (also known as the heat storage). The specific calculation procedures for above fluxes are presented in previous work.^53^ Q_A_, the term of anthropogenic heat release, is estimated individually here based on a spatial proxy from DMSP/OLS nighttime light intensity,^78^ since which contains the lights from cities, towns, and other sites with persistent lighting, including gas flares, demonstrating uniquely superior in characterizing human activity and the spatial distribution of anthropogenic thermal energy.

#### Space-for-time strategy and temporal analysis

Elements such as population density, vegetation coverage, and ISP distribution possess spatial gradient patterns in urban areas.^1^^,^^79^ Space-for-time strategy provides new insight for estimating and projecting urbanization effects based on a straightforward linear regression between LST (or SEB terms) and ISP gradient across space.^8^^,^^32^^,^^74^ Estimated changes of LST and SEB flux could be quantified by establishing the linear regression models between ISP and surface variables, given by:

(Equation 3) $${\text{LST}}_{i}=k\times {\text{ISP}}_{i}+b\;\left(i=1,2,......,n\right)$$

(Equation 4) $${\text{SEB}}_{i}=k\times {\text{ISP}}_{i}+b\;\left(i=1,2,......,n\right)$$

where *k* is regression coefficient and *b* is constant, computed by the ordinary least square (OLS) regression, and *n* represents valid 0.05^o^ grids within each of moving windows. Then both Lilliefors test and Jarque-Bera test (which are regular functions in MATLAB, Mathworks) are performed for the normal distribution test in regression fittings of ISP and surface variables. For most of variables, more than 70% of statistical windows pass the tests, demonstrating the rationality of spatial gradient methods and linear regression fitting between ISP and the climatic variables.

Outcomes demonstrate that the potential changes in any of the variables at both annual and seasonal scales when ISP increases 1% (0.05^o^) locally based on the space-for-time substitution behave very consistent from 2003 to 2018. The average value and median value (of 16 years) are close for the estimations in each of variables. The relatively stable statistics over time further confirm the robustness of linear relationships between ISP and all variables (LST and SEB).^32^^,^^74^

We conduct urban-induced warming estimates at regional scale in the major urbanized regions worldwide which are chosen according to full consideration of urban impervious surfaces distribution, country geography, effective statistical data. They include Eastern United States, Europe, Eastern China, Northeast China, India, Western Russia, Japan and South Korea region, Southeast Asia region, and Gulf of Guinea, West Africa.

On the one hand, the potential changes of temperature and SEB flux due to urbanization hypothesizing the process of land surface from natural watershed (ISP = 0) to high degree of urbanization (ISP = 50%) are evaluated across these regions, following the method described in previous work.^53^ On the other hand, actual surface warming or amplitudes in all variables induced by urbanization during the period 2003-2018 are quantified individually. The specific changes are calculated by the production of potential change due to increasing ISP by 1% on the basis of the space-for-time approximation, and actual ISP increment over the study period (Figure S8) which is calculated by fitting four functions encompassing linear, quadratic, cubic and exponential, combined the corrected Akaike Information Criterion AICc^80^ since non-linear growth of ISP across most urbanized locations worldwide has been detected.

Furthermore, we adopt temporal analysis on urbanization effects in the major urbanized regions over the same study period. As proposed elsewhere,^35^^,^^81^ observed large-scale climatic trend for temperature during a given period is generally forced by urbanization signal and natural climate variability. We therefore isolate temporal urbanization effects on LST and SEB via the difference of changing trends between all sample sites and rural sites in each statistical window corresponding to above space-based analysis:

(Equation 5) $$\Delta {Trend}_{\text{urbanization}}=Trend\;\left(\text{Variabl}e_{\text{all}\;\text{samples}}-\text{Variabl}e_{\text{rural}\;\text{sites}}\right)$$

Where rural sites are defined as ISP < 1% (on 0.05^o^ grids) during 2003-2018.

We perform sensitivity tests for the multiple search windows (0.5^o^-by-0.5^o^ used in this paper; 1.0^o^-by-1.0^o^, 1.5^o^-by-1.5^o^, 2.0^o^-by-2.0^o^) in space-for-time substitution (Equations 3 and 4), as well as threshold definitions (ISP < 1% used in this paper; ISP < 0.1%, 2%, 5%; and ΔISP < 0.5%, 2%) in temporal analysis (Equation 5). We find consistent outcomes by applying various window strategies which consolidate the robustness of space-for-time in quantifying urbanization effects. The selection of ISP <1% is in general reasonable in balancing statistical significance and the representative of rural background, given more urbanization signal would be blended in when increasing ISP threshold.

#### Estimations of urban warming bias and SUHI footprint

We examine the warming bias triggered by urbanization through the comparison in LST trends between global and rural grids delineated globally from 2003 to 2018. Rural samples are defined according to ISP threshed (< 1% on 0.5^o^ raster grid), shown in Figure 5A. Then the linear regression equation is built to calculate the climatic variability in two LST series. To eliminate the latitudinal and altitude effects of LST, we conduct an anomaly algorithm to LST series via subtracting the average of 16 years.

The FP of SUHI effect refers to the spatial extent of SUHI, implying that urban warming is not limited to its city boundary, but extends to vicinities over a certain distance. Previous researches have reported the FP of SUHI effect that is several times of urban size in regional extent,^43^^,^^44^ which is estimated here at global scale. Specifically, we conduct the estimations of SUHI footprint and areas according to the fact that a gradual decrease of the sliding slope in LST from city center to rural counterparts is observed, using global 1,860 cities with a population larger than 300,000.^12^ The footprint of SUHI can be estimated by the following three steps.

1. Based on the section above (see “Calculation of SUHI intensity and surface energy balance”), we extract the overall urban-rural domain coving all grids from city center to the farthest rural point (except for water bodies or elevation ±50 m above/below urban areas) for 536 large cities. Temperature anomalies (through subtracting the average of LST in rural areas) and actual distance (km) to city center (using a conversion of latitude/longitude to actual distance; Equation 6) for each grid are then counted.

(Equation 6) $$D=\arccos\left[\cos\left(Y1\right)\cos\left(Y2\right)\cos\left(X1-X2\right)+\sin\left(Y1\right)\sin\left(Y2\right)\right]\times R_{\text{earth}}$$

Where D is actual distance between two geographic locations with latitude/longitude coordinates (X1, Y1) and (X2, Y2), respectively; R_earth_ represents the average radius of the Earth (equals to 6,371.393 km).

2. To investigate the variation of temperature anomaly from city center to rural in 536 large cities (for instance, in 2018), we obtain averaged LST sequence that varies with distance during interval of 1 km, then calculate the sliding slope (°C/km) of the sequence (set sliding statistic length *n* = 15). We find that in general, the slope exhibits negative but decreasing from city center to rural, then close to zero, suggesting that the spatial impact SUHI effect is limit, thus SUHI FP exists for large cities. We quantify the FP value by definition that absolute value of sliding slope of 10 consecutive sequences is less than 0.01°C/km since one location. Then four types of the cities are classified according to population gradient (1,000–2,000, 2,000–4,000, 4,000–10,000, and 10,000–40,000 K) and corresponding FP distances annually from 2003 to 2018 are estimated via repeating the above operations.
3. Further, we conduct a maximization evaluation of global FP areas of SUHI effect using 1860 cities with a population exceeding 300 K in 2018. We adopt the median of FP values estimated during 16 years for each population gradient and use the FP for 1,000–2,000 population as FP value for cities with < 1,000 population, for the sake of maximization assessment of global FP area. The FP spatial extents (km^2^) of SUHI effect at global scale are computed based on the circular area with radius of corresponding FP distance in 1860 cities (Figure S16).
