# Supplementary material for: Intensifying urban imprint on land surface warming: Insights from local to global scale
Source: iScience. 2024 Feb 5;27(3):109110. doi: 10.1016/j.isci.2024.109110 (PMC10904926; doi:10.1016/j.isci.2024.109110)
Supplement: Document S1. Figures S1–S16 and Tables S1 and S2 [file mmc1.pdf]

iScience, Volume *27*

Supplemental information

Intensifying urban imprint on land surface warming: Insights from local to global scale

Pengke Shen and Shuqing Zhao

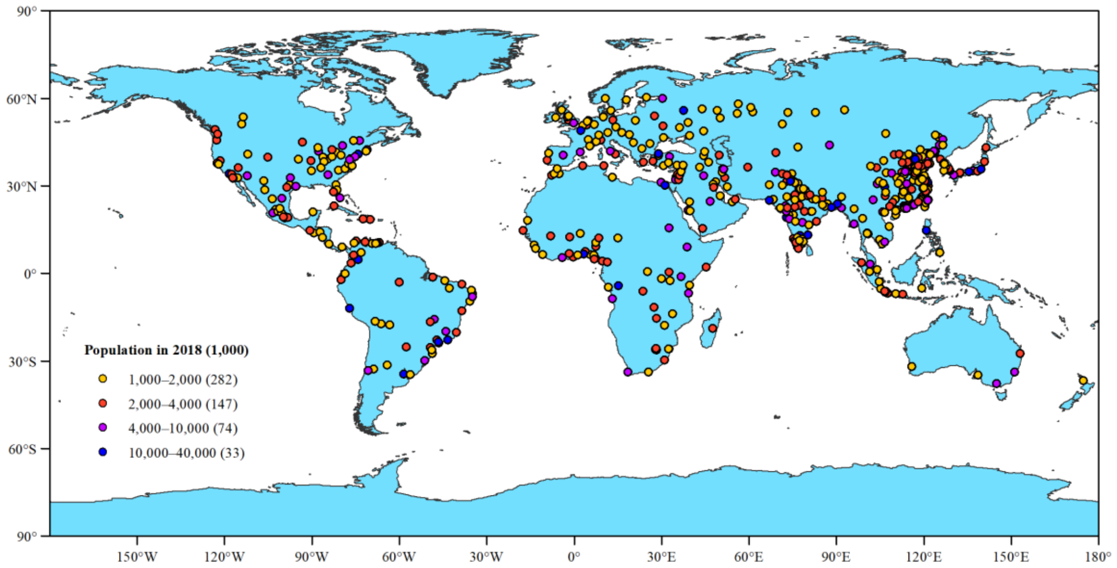

Figure S1. Global 536 large cities and population distribution in 2018. Related to Figure 1 and STAR Methods.

The large cities are divided into four categories according to the population gradients (1,000–2,000 K, 2,000–4,000 K, 4,000–10,000 K, 10,000–40,000 K), shown in different colors.

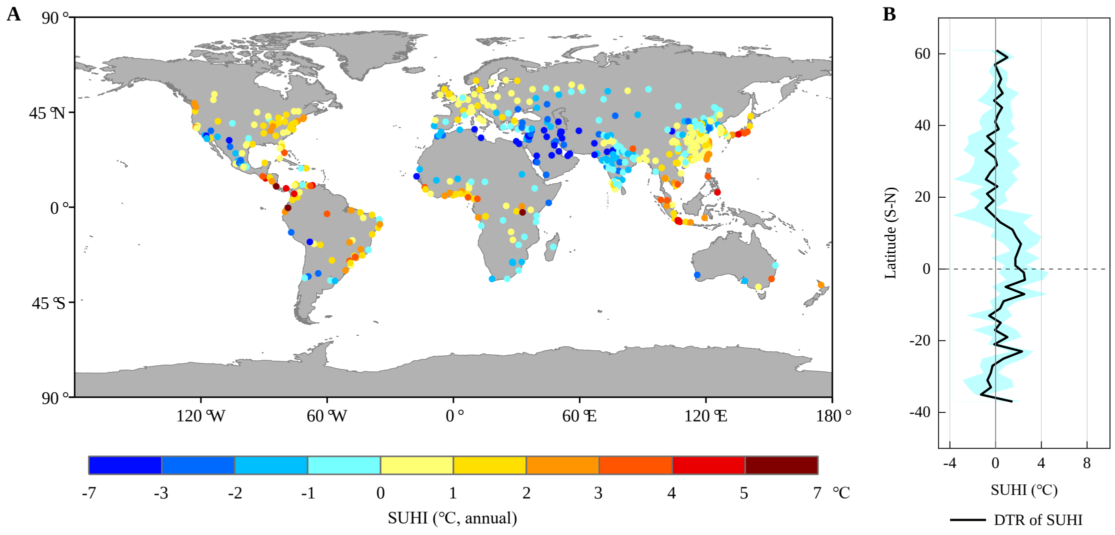

Figure S2. Spatial distribution and latitudinal average of annual DTR of SUHI intensity averaged over the period 2003−2018 across 536 global large cities. Related to Figure 1.

(A), Spatial distribution of DTR intensity (unit: °C). (B), Shaded area shows 5%–95% range of the DTR of SUHI.

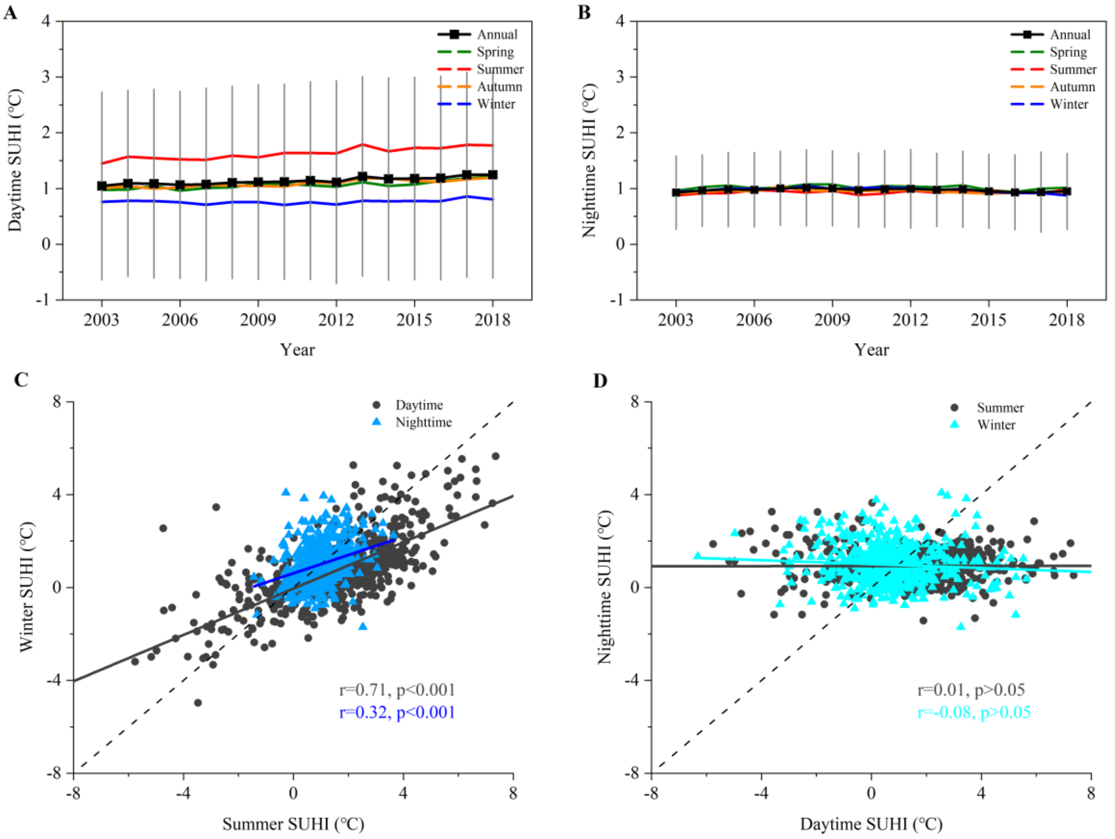

Figure S3. Temporal variations of SUHI intensity (℃) for 536 large cities from 2003-2018. Related to Figure 1 and Figure 2.

(A-B), Inter-annual change of annual and seasonal SUHI intensities in daytime and nighttime, respectively. Error bars represent the standard deviation (SD) of annual SUHI. (C-D), Relationships of SUHI for all cities between summer and winter, and between daytime and nighttime, respectively. Linear fittings are based on ORTH regression with Pearson correlation test.

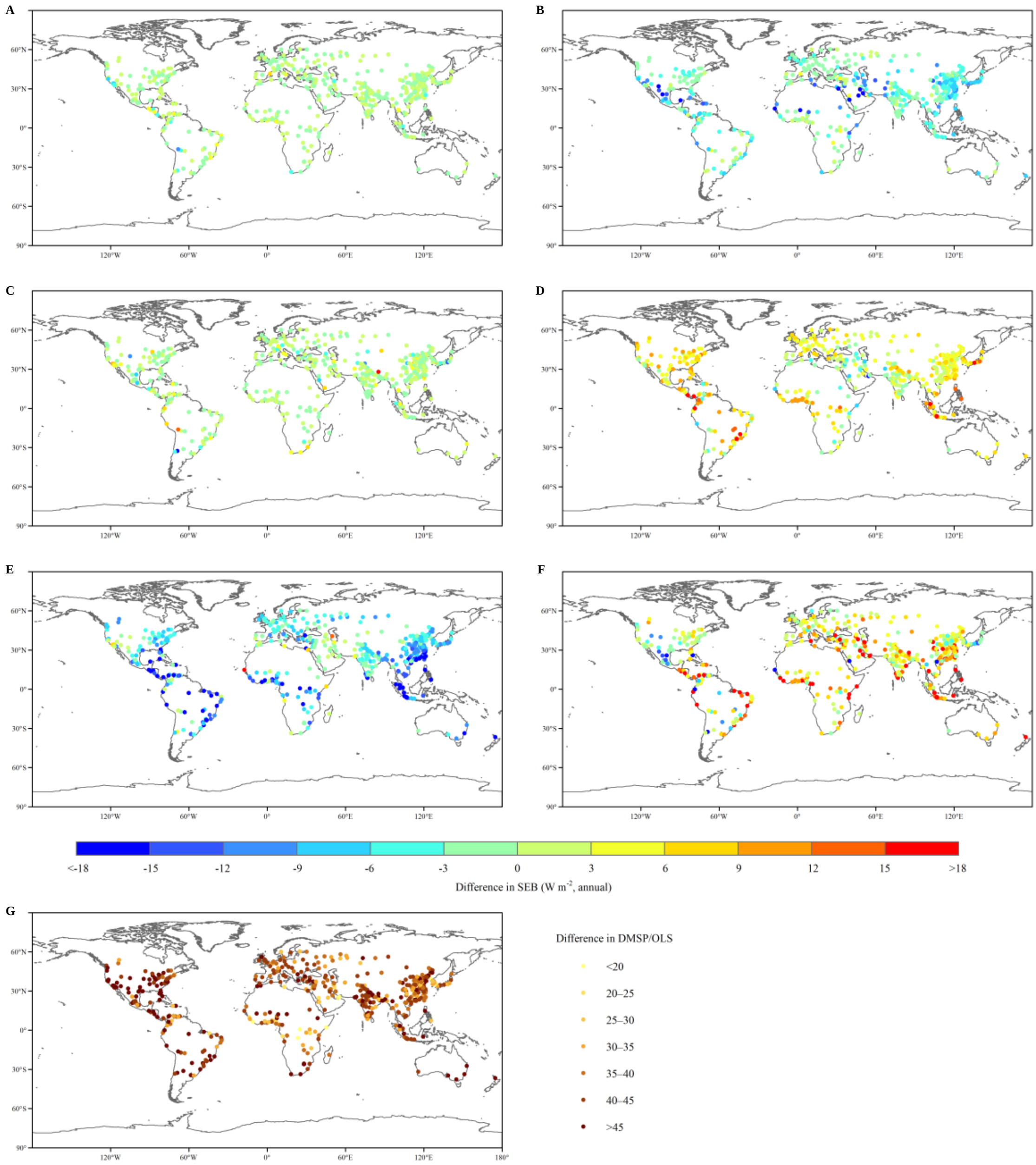

Figure S4. Spatial distribution of difference in annual mean surface energy fluxes between urban and rural areas averaged over the period 2003-2018 across 536 global big cities and differences. Related to Figure 2.

(A) SWd, (B) SWu, (C) LWd, (D) LWu, (E) LE, (F) H+G (W m^{-2}); And (G) DMSP/OLS.

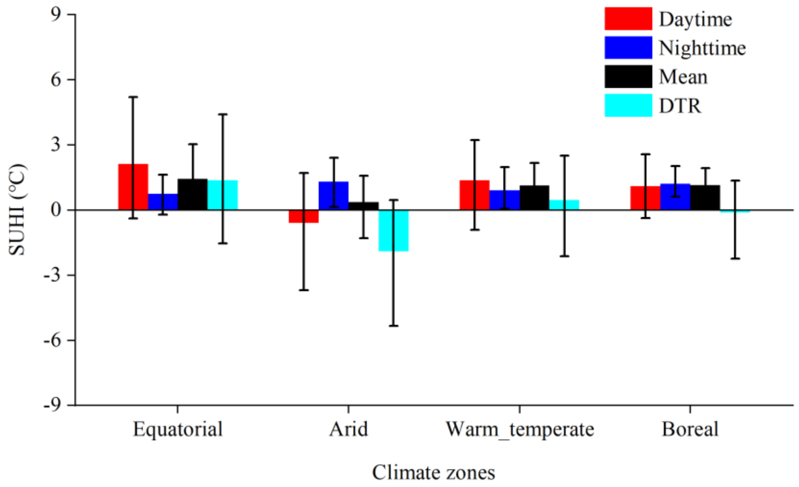

Figure S5. Comparison of annual SUHI among major climate zones. Related to Figure 2. Climate zones include equatorial, arid, warm temperate and boreal climates. Error bars encompass the 5–95% range.

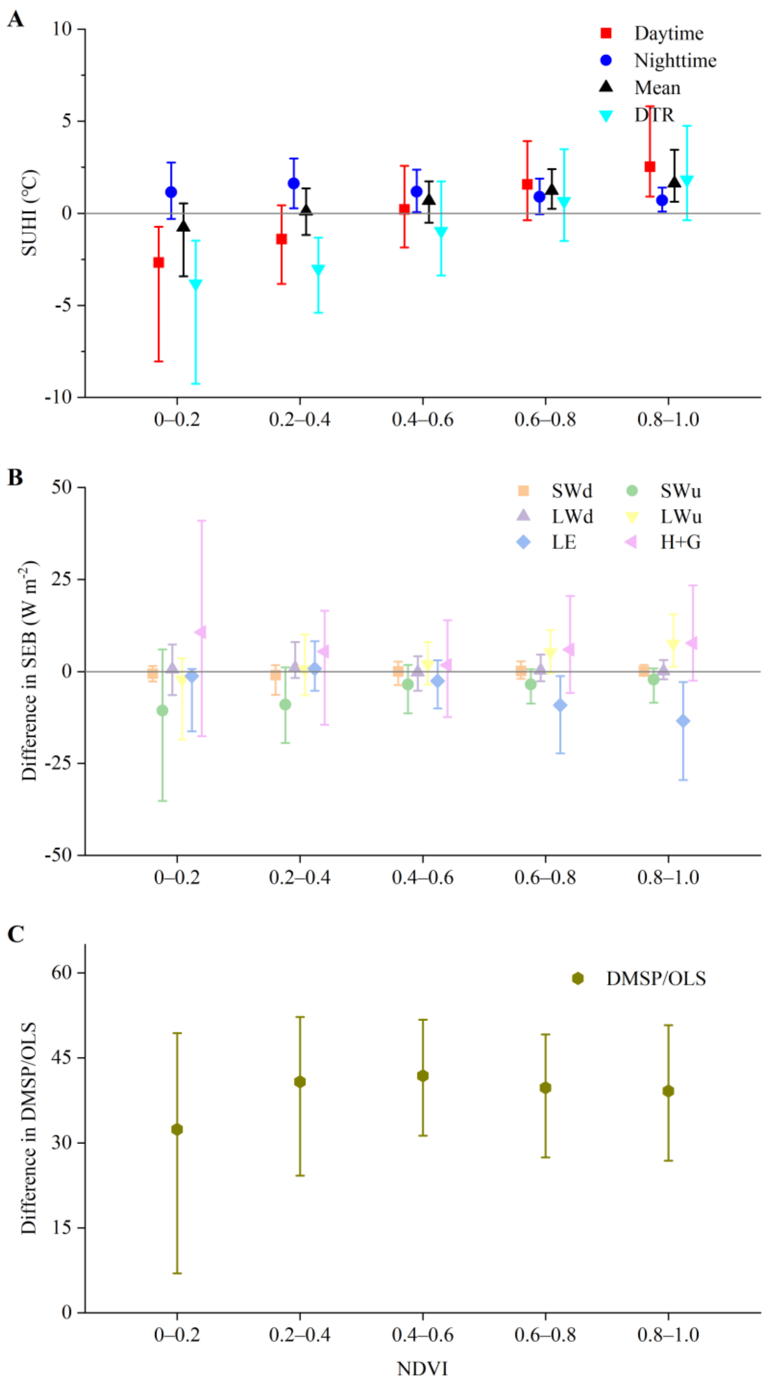

Figure S6. Relationship between annual SUHI, difference in SEB, DMSP/OLS (between urban and rural areas) and multi-year average of NDVI. Related to Figure 2.

(A), SUHI (℃); (B), Difference in SEB (W m^{-2}); (C), Difference in DMSP/OLS. The NDVI intervals include 0–0.2, 0.2–0.4, 0.4–0.6, 0.6–0.8, and 0.8–1.0. Error bars encompass the 5–95% range.

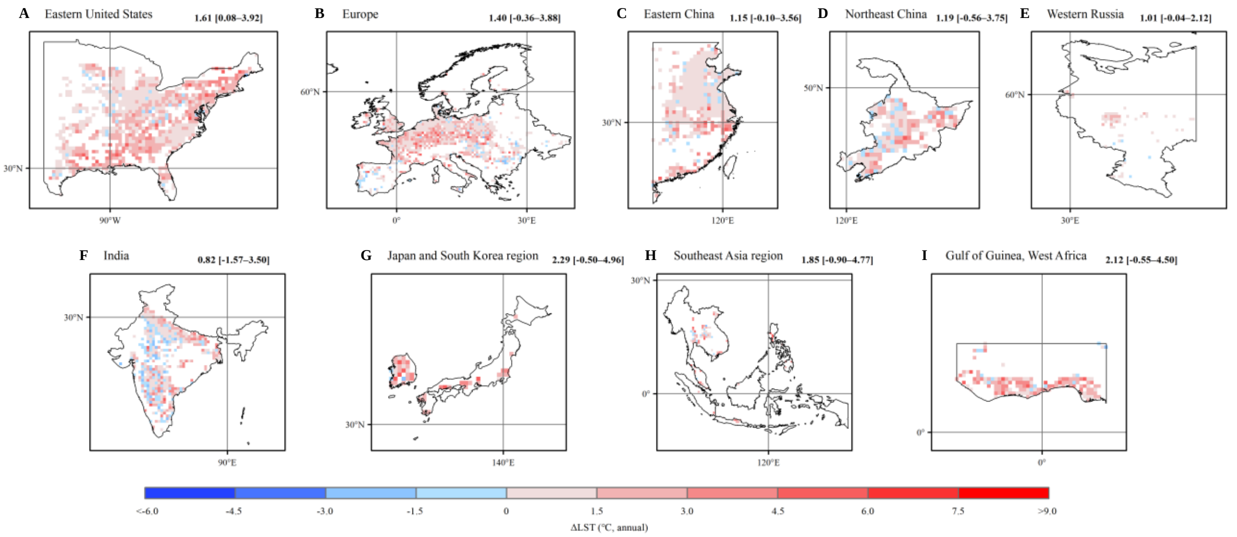

Figure S7. Potential change in annual mean LST caused by urbanization in the nine major urbanized regions, spanning 2003-2018. Related to Figure 3A.

The quantifications are conducted by space-for-time substitution assumed 50% increase in ISP for 0.5° grids (METHOD DETAILS). (A-I), Eastern United States, Europe, Eastern China, Northeast China, India, Western Russia, Japan and South Korea region, Southeast Asia region, and Gulf of Guinea, West Africa. Raster resolution of 0.5° in all plots.

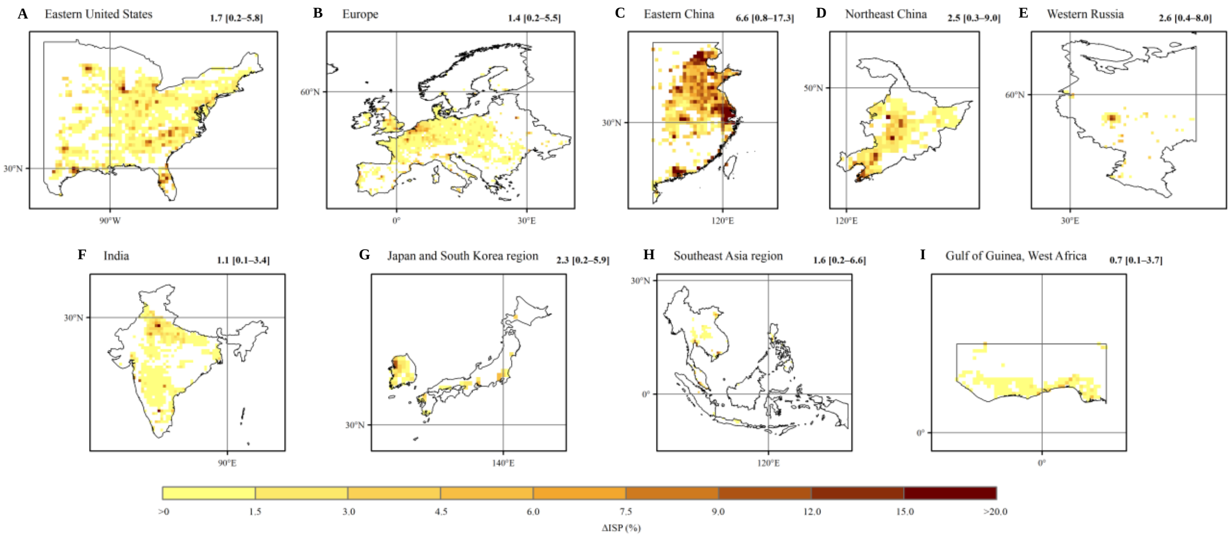

Figure S8. Actual increment of ISP in the nine major urbanized regions from 2003-2018. Related to STAR Methods.

ΔISP is calculated by four regression fitting models and the corrected Akaike Information Criterion (AICc) (METHOD DETAILS). The resolution of the grid points in all figures is 0.5°.

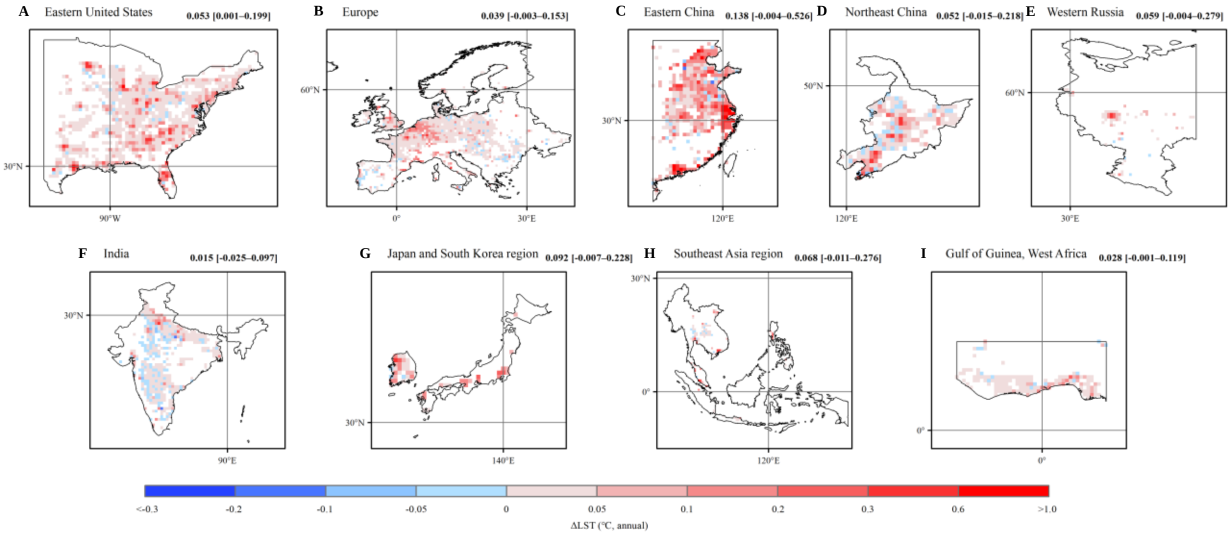

Figure S9. Space-for-time estimates in annual mean LST caused by urbanization in the nine major urbanized regions, spanning 2003-2018. Related to Figure 3B.

The quantifications are conducted by space-for-time substitution and actual increment in ISP (Figure S8). Raster resolution of 0.5^{o} in all plots.

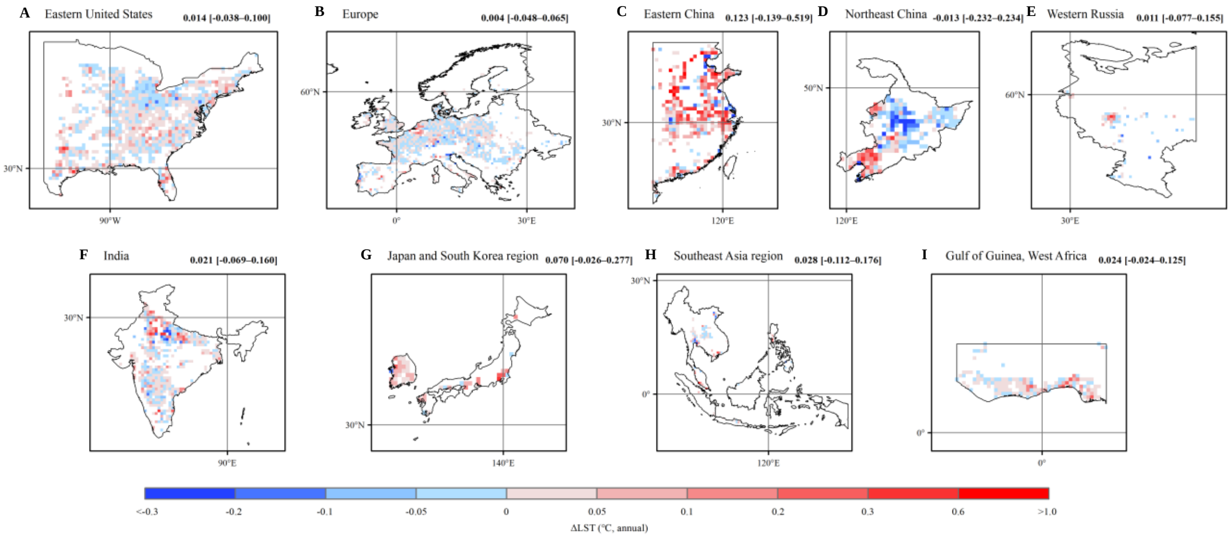

Figure S10. Potential change in annual mean LST caused by urbanization in the nine major urbanized regions, spanning 2003-2018. Related to Figure 3C.

The quantifications are conducted by temporal analysis of urbanization effect (METHOD DETAILS). Raster resolution of 0.5° in all plots.

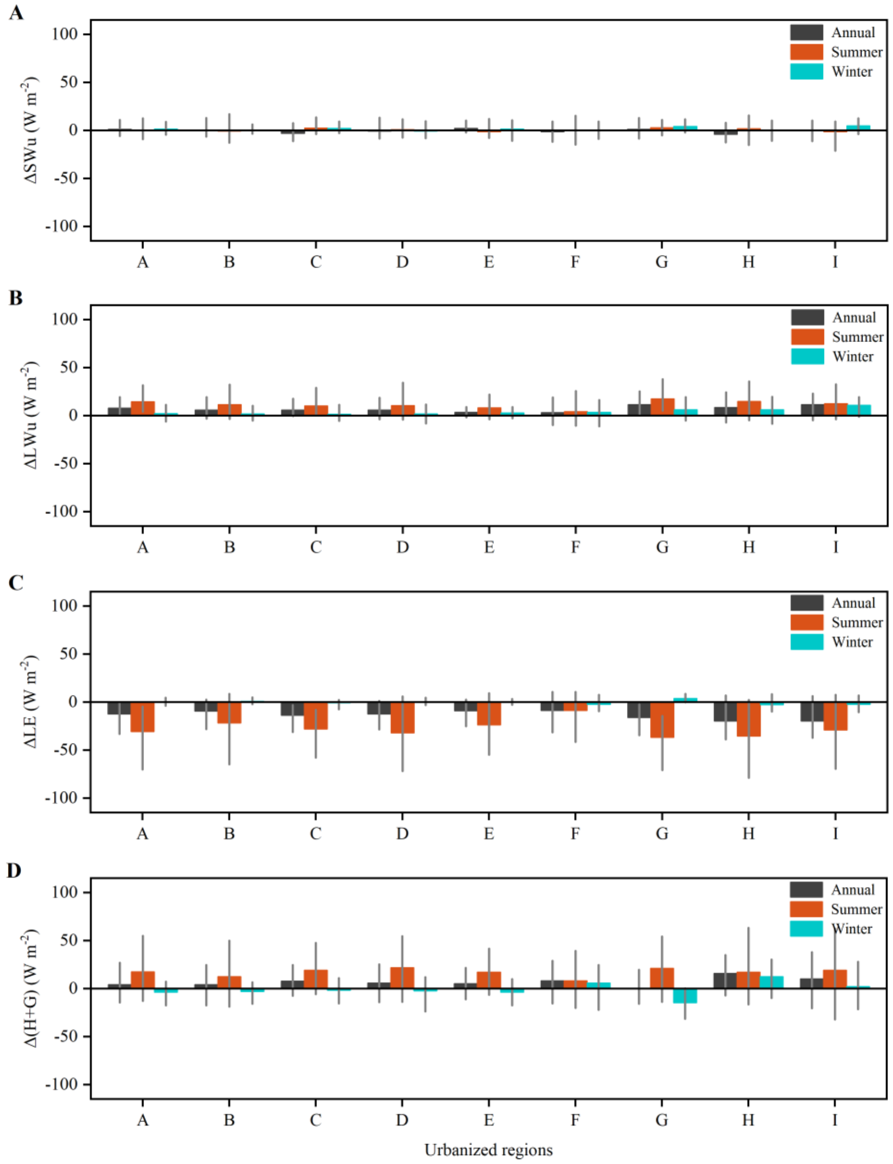

Figure S11. Potential change in surface energy flux perturbation in the nine major urbanized regions (assumed 50% increase in ISP for 0.5° statistical window), 2003-2018 (based on spatial gradient analysis method). Related to Figure 3.

(A-D), SWu, LWu, LE, and H+G, respectively. Histogram and error lines represent the mean and 5–95% range. A-I of X axis represent the nine major urbanized regions, i.e., Eastern United States, Europe, Eastern China, Northeast China, Japan and South Korea region, Western Russia, India, Southeast Asia region, and Gulf of Guinea, West Africa, respectively.

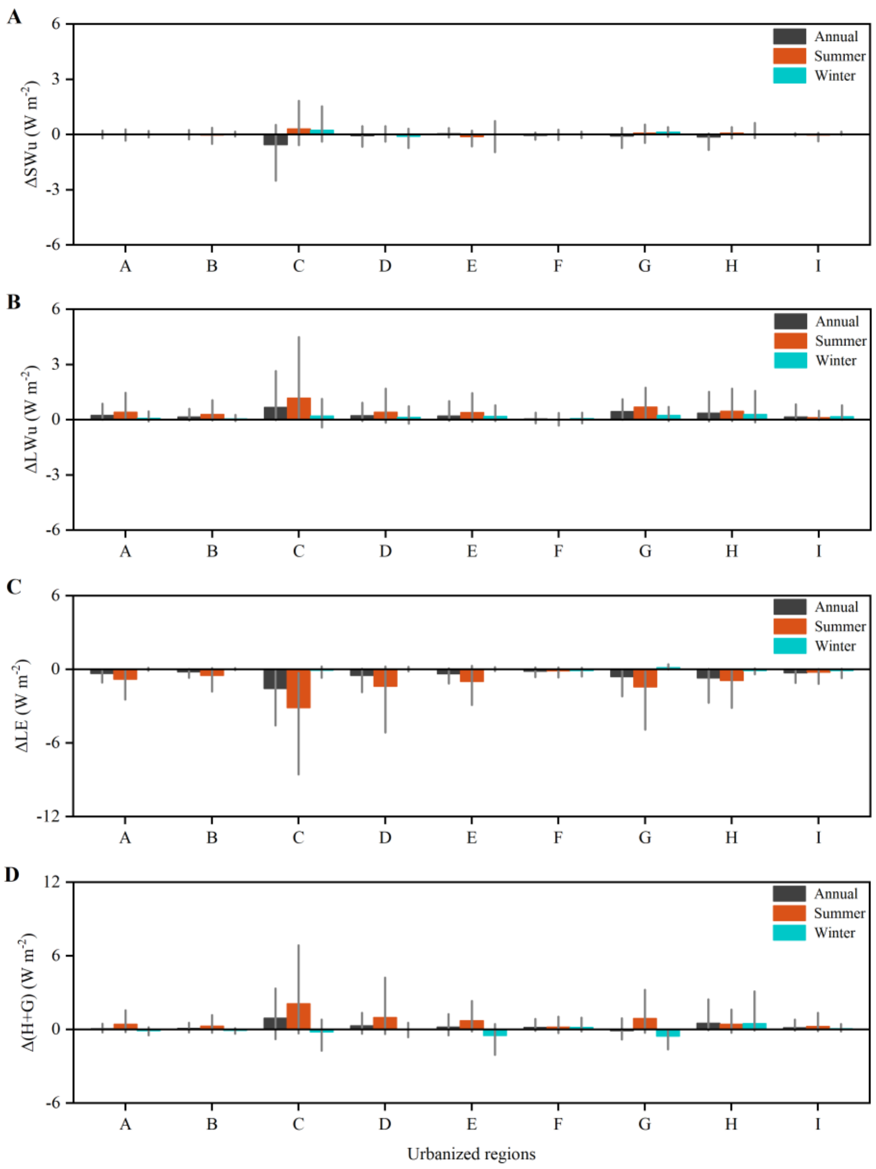

Figure S12. Actual changes in surface energy flux perturbation in the nine major urbanized regions, spanning 2003-2018 (based on spatial gradient analysis method and actual increment in ISP). Related to Figure 3.

(A-D), SWu, LWu, LE, and H+G, respectively. Histogram and error lines represent the mean and 5–95% range. A-I of X axis represent the nine major urbanized regions.

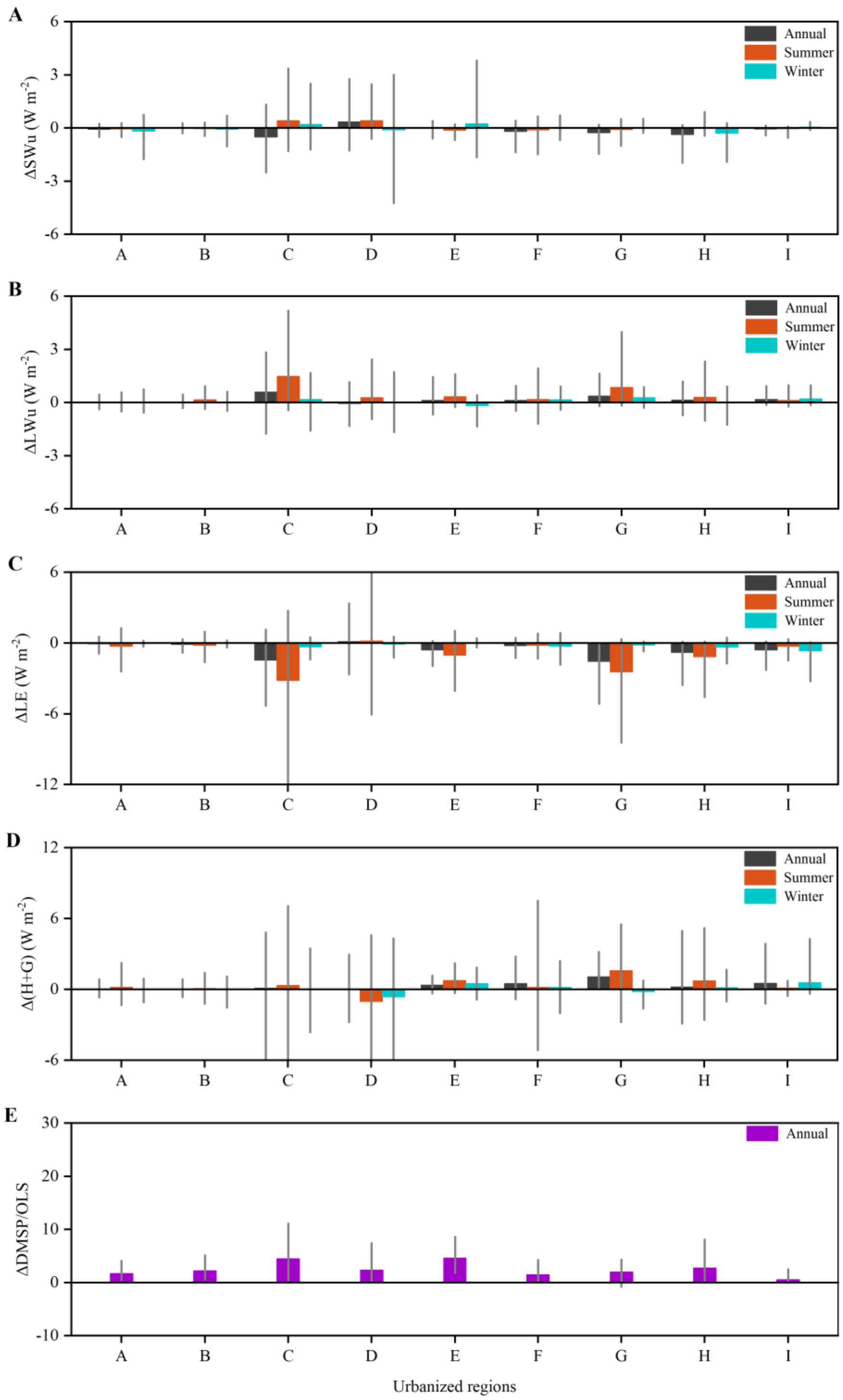

Figure S13. Same as Figure S12, but based on temporal analysis method. Related to Figure 3.

(A-D), SWu, LWu, LE, and H+G, respectively. (E), DMSP/OLS.

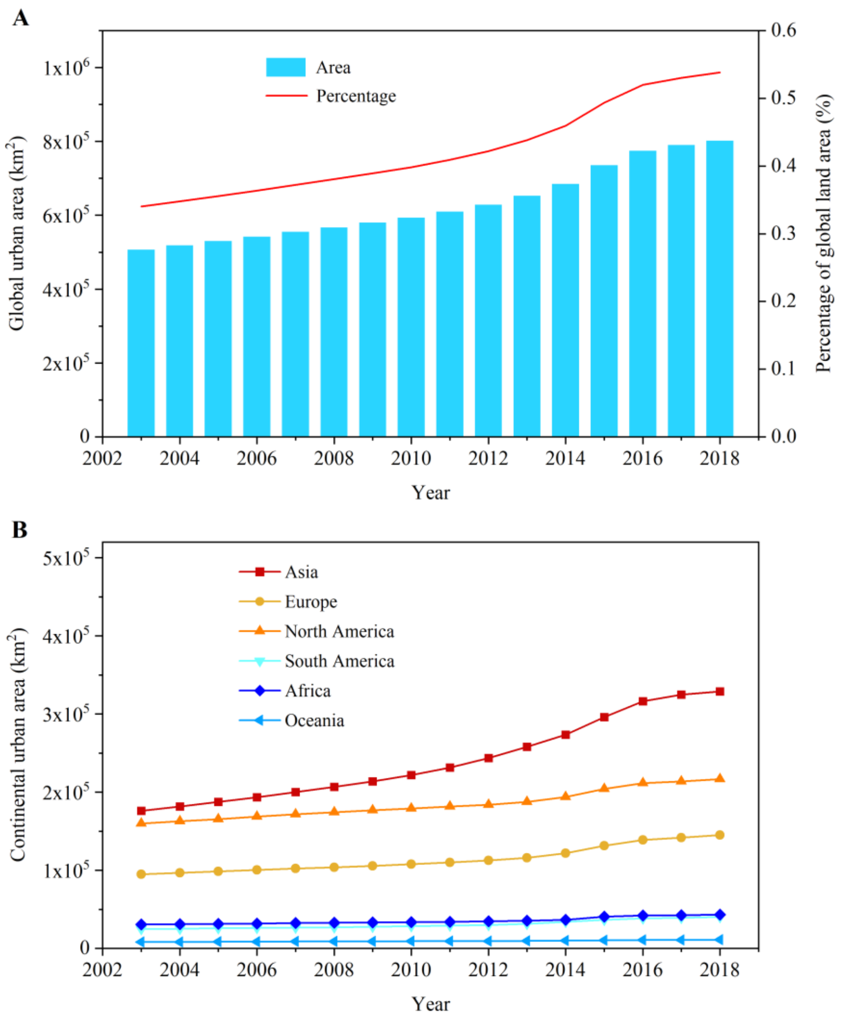

Figure S14. Temporal dynamics of global urban impervious surfaces area from 2003-2018. Related to Figure 5.

(A), Global; (B), Six continents (i.e., Asia, Europe, North America, South America, Africa, and Oceania).

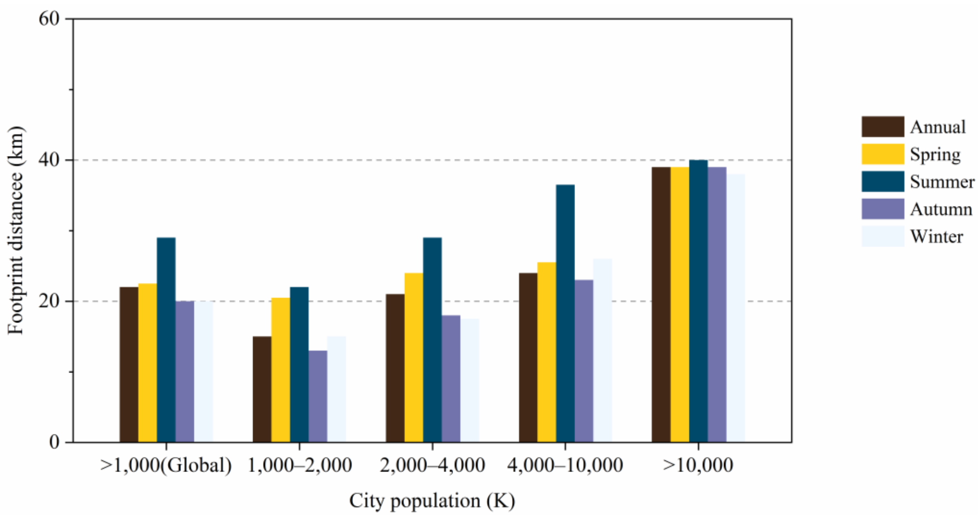

Figure S15. Annual and seasonal FP values calculated from the median during period 2003-2018. Related to Figure 6.

Global 536 large cities are classified as four categories based on city population 1,000–2,000 K (282 cities), 2,000–4,000 K (147 cities), 4,000–10,000 K (74 cities), and 10,000–40,000 K (33 cities).

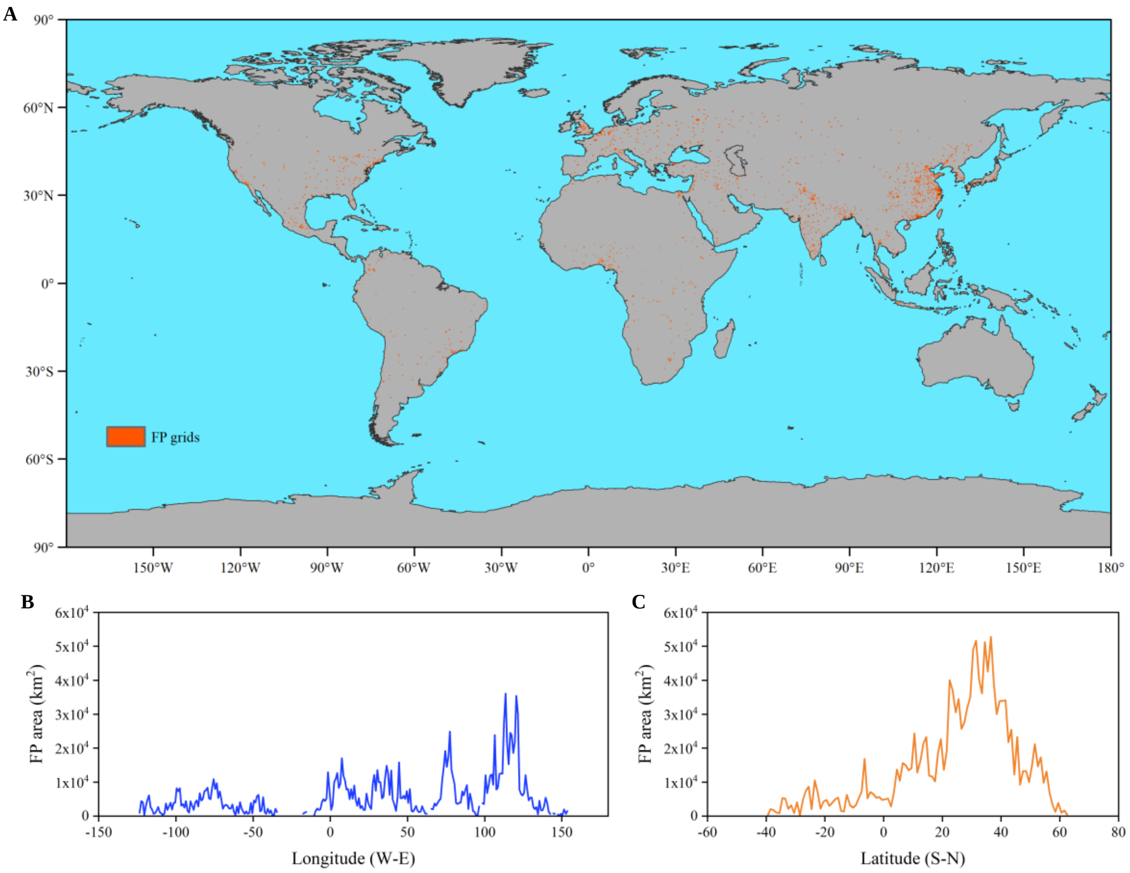

Figure S16. Global footprint distribution of annual SUHI effect for 1,860 cities with population more than 300K (in 2018) during 2003-2018. Related to Figure 6 and STAR Methods.

(A), The footprint grids are shown as red color (with resolution 0.05°) on the above plot. (B-C), The subplots below show the distribution of annual FP areas per 1° longitude/latitude bin.

Table S1. Spatial Pearson correlation coefficients between seasonal SUHI intensities and SEB variances (between urban and rural areas) across 536 global big cities, averaged from 2003-2018. Related to Figure 2.

***, ** and * represent the correlation coefficient is significant at 0.001, 0.01 and 0.05 level, respectively.

		SEB variances between urban and rural areas						
		**SWd**	**SWu**	**LWd**	**LWu**	**LE**	**H+G**	**DMSP /OLS**
Daytime	Spr.	0.14**	0.47***	0.02	0.89***	-0.54***	0.01	0.10*
	Sum.	0.13**	0.48***	0.04	0.92**	-0.64***	0.01	0.15***
	Aut.	0.20***	0.49***	0.01	0.88***	-0.57***	-0.04	0.15***
	Win.	0.23***	0.34***	0.01	0.80***	-0.49***	0.07	0.14**
Nighttime	Spr.	-0.08	-0.32***	0.02	0.18***	0.16***	-0.15***	0.13**
	Sum.	0.01	-0.28***	0.06	0.30***	-0.08	0.06	0.10*
	Aut.	-0.07	-0.38***	0.13**	0.17***	0.09*	0.05	0.14**
	Win.	-0.02	-0.39***	0.13**	0.41***	0.05	-0.03	0.10*
Mean	Spr.	0.11*	0.34***	0.03	0.95***	-0.48***	-0.05	0.15***
	Sum.	0.12**	0.38***	0.05	0.97***	-0.64***	0.02	0.17***
	Aut.	0.17***	0.35***	0.07	0.95***	-0.54***	-0.02	0.21***
	Win.	0.20***	0.12**	0.08	0.93***	-0.42***	0.05	0.18***
DTR	Spr.	0.15***	0.52***	0.01	0.72***	-0.53***	0.05	0.04
	Sum.	0.12**	0.55***	0.02	0.79***	-0.59***	-0.01	0.11*
	Aut.	0.19***	0.56***	-0.03	0.71***	-0.53***	-0.06	0.09*
	Win.	0.21***	0.47***	-0.05	0.49***	-0.44***	0.07	0.07

Table S2. Estimation of footprint (FP) area of SUHI effect for global 1,860 cities during 2003-2018. Related to Figure 6.

The unit of FP area is km^2, and *PCT* denotes the proportion of FP area to global land area or each continent.

	Asia	Europe	North America	South America	Africa	Oceania	**Global**
Number of cities	1014	239	233	137	222	15	**1860**
Annual	8.22×10^5	1.75×10^5	1.90×10^5	1.16×10^5	1.71×10^5	0.10×10^5	$\mathbf{1.48\times10^6}$
PCT	1.87%	1.75%	0.79%	0.64%	0.57%	0.11%	**1.00%**
Spring	1.35×10^6	3.04×10^5	3.13×10^5	1.85×10^5	2.81×10^5	0.15×10^5	$\mathbf{2.45\times10^6}$
PCT	3.07%	3.04%	1.30%	1.03%	0.94%	0.17%	**1.64%**
Summer	1.65×10^6	3.62×10^5	3.91×10^5	2.23×10^5	3.47×10^5	0.20×10^5	$\mathbf{2.99\times10^6}$
PCT	3.75%	3.62%	1.63%	1.24%	1.16%	0.22%	**2.01%**
Autumn	6.48×10^5	1.35×10^5	1.50×10^5	0.93×10^5	1.34×10^5	0.08×10^5	$\mathbf{1.17\times10^6}$
PCT	1.47%	1.35%	0.62%	0.52%	0.45%	0.09%	**0.78%**
Winter	8.01×10^5	1.73×10^5	1.85×10^5	1.11×10^5	1.64×10^5	0.10×10^5	$\mathbf{1.44\times10^6}$
PCT	1.82%	1.73%	0.77%	0.62%	0.55%	0.11%	**0.97%**
